# Genetic Variation in Low-To-Medium-Affinity Fcγ Receptors: Functional Consequences, Disease Associations, and Opportunities for Personalized Medicine

**DOI:** 10.3389/fimmu.2019.02237

**Published:** 2019-10-03

**Authors:** Sietse Q. Nagelkerke, David E. Schmidt, Masja de Haas, Taco W. Kuijpers

**Affiliations:** ^1^Sanquin Research and Landsteiner Laboratory, Department of Blood Cell Research, Amsterdam UMC, University of Amsterdam, Amsterdam, Netherlands; ^2^Pediatric Hematology, Immunology and Infectious Diseases, Emma Children's Hospital, Amsterdam UMC, University of Amsterdam, Amsterdam, Netherlands; ^3^Sanquin Research and Landsteiner Laboratory, Department of Experimental Immunology, Amsterdam UMC, University of Amsterdam, Amsterdam, Netherlands; ^4^Sanquin Diagnostic Services, Department of Immunohematology Diagnostics, Amsterdam, Netherlands; ^5^Sanquin Research, Center for Clinical Transfusion Research, Leiden, Netherlands; ^6^Jon J. van Rood Center for Clinical Transfusion Science, Leiden University Medical Center, Leiden, Netherlands; ^7^Department of Immunohematology and Blood Transfusion, Leiden University Medical Center, Leiden, Netherlands

**Keywords:** Fc gamma receptor (FcγR), genetic variation, autoinflammatory and autoimmune diseases, immunotherapy, mechanisms of disease

## Abstract

Fc-gamma receptors (FcγR) are the cellular receptors for Immunoglobulin G (IgG). Upon binding of complexed IgG, FcγRs can trigger various cellular immune effector functions, thereby linking the adaptive and innate immune systems. In humans, six classic FcγRs are known: one high-affinity receptor (FcγRI) and five low-to-medium-affinity FcγRs (FcγRIIA, -B and -C, FcγRIIIA and -B). In this review we describe the five genes encoding the low-to-medium -affinity FcγRs (*FCGR2A, FCGR2B, FCGR2C, FCGR3A*, and *FCGR3B)*, including well-characterized functionally relevant single nucleotide polymorphisms (SNPs), haplotypes as well as copy number variants (CNVs), which occur in distinct copy number regions across the locus. The evolution of the locus is also discussed. Importantly, we recommend a consistent nomenclature of genetic variants in the *FCGR2/3* locus. Next, we focus on the relevance of genetic variation in the *FCGR2/3* locus in auto-immune and auto-inflammatory diseases, highlighting pathophysiological insights that are informed by genetic association studies. Finally, we illustrate how specific FcγR variants relate to variation in treatment responses and prognosis amongst autoimmune diseases, cancer and transplant immunology, suggesting novel opportunities for personalized medicine.

## Introduction

Fc-gamma receptors (FcγR) are cellular receptors for Immunoglobulin G (IgG) and upon binding of complexed IgG can trigger various cellular immune effector functions that can destroy and eliminate the opsonized target. These responses are only initiated when multiple IgG molecules are bound simultaneously; a single IgG does not activate FcγRs. When multiple IgG molecules are fixed close to each other, as is the case in opsonized bacteria or in an immune complex, this results in cross-linking of several FcγRs in the cell membrane, which leads to their activation. In this way, FcγRs play an important role in immunity by linking the adaptive and innate immune systems.

**Figure 1:**
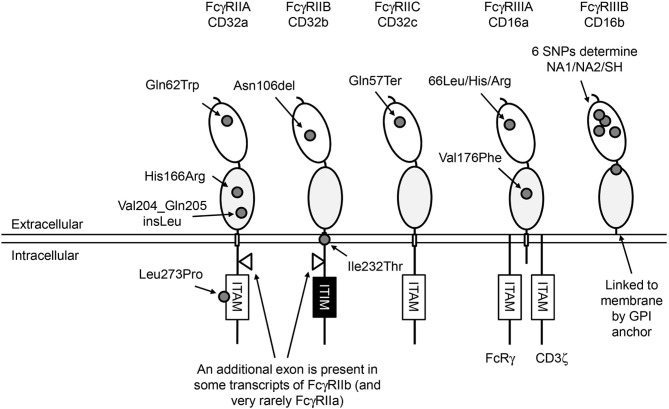
Human FcγRs. Overview of the structure of human low-to-medium-affinity FcγRs. The oval shapes in the extracellular part of the FcγRs represent the different extracellular domains; the light gray domains are the domains where IgG molecules bind. All FcγRs except FcγRIIIB are linked to the plasma membrane by transmembrane (TM) domains indicated by small rectangles. FcγRIIIB is linked to the plasma membrane through a GPI anchor. FcγRIIIA has a small intracellular domain that associates with adaptor molecules that can initiate an intracellular signaling cascade when multiple FcγRs are cross-linked, which ultimately leads to activation of the cell on which the FcγRs are expressed. FcγRII receptors have a much larger intracellular domain, and contain a signaling motif to start this cascade in their own polypeptide chain. Signaling by activating FcγRs is mediated by immunoreceptor tyrosine-based activating motifs (ITAM) that are present either in the cytoplasmic tail of the receptor itself or in non-covalently associated signaling adaptor proteins, such as the common γ-chain (FcRγ). Aggregation of activating FcγR by binding of multivalent ligands, such as an opsonized pathogen or blood cell or an immune complex, results in the phosphorylation of ITAM tyrosine residues by Src family protein tyrosine kinases (PTKs), and ultimately leads to activation of cellular responses (1). Aggregation of the inhibitory FcγRIIB, which contains an immunoreceptor tyrosine-based *inhibitory* motif (ITIM), also results in phosphorylation of tyrosine residues by Src family PTKs. In contrast to ITAMs, phosphorylated ITIMs serve as binding sites for phosphotyrosine phosphatases (PTPs) which dephosphorylate other proteins resulting in inhibition of activating pathways (2). Approximate location of functional SNPs in the FcγRs are indicated by small gray circles, SNPs are indicated by 3-letter amino-acid codes. ITAM, immunoreceptor tyrosine-based activating motif; ITIM, immunoreceptor tyrosine-based inhibitory motif.

FcγRs are differentially expressed on a range of immune cells (Figure 1) (3). In humans, six classic FcγRs are known, which can be distinguished into one high-affinity receptor (FcγRI) and five low-to-medium-affinity FcγRs (FcγRIIA, -B and -C, FcγRIIIA and -B). The low-to-medium-affinity FcγRs are the focus of this review. On a functional level, most of the FcγRs are activating receptors that can induce the cellular responses mentioned above, but one, FcγRIIB, is an inhibitory receptor.

The five genes for the low-to-medium-affinity FcγRs are located in a single cluster on chromosome 1q23.3 (the *FCGR2/3* locus) and several genetic variations resulting in functional changes have been found in all of the genes in this locus. These variations are associated with auto-immune, auto-inflammatory, and infectious diseases and with efficacy of immunotherapy in cancer patients, but genetic analysis of the variants at the locus is hampered by the genetic complexity deriving from a segmental duplication, inconsistent nomenclature, and a high degree of linkage disequilibrium.

The gene encoding FcγRI, *FCGR1A*, is also located at chromosome 1 (1q21.2), and has two presumed pseudogenes (*FCGR1B* at 1p11.2 and *FCGR1C* at 1q21.1) that have stop codons in the third extracellular domain and theoretically cannot be expressed as transmembrane receptors (4). Recently, some functional SNPs that occur at low frequency in the population were discovered in *FCGR1A* (5, 6), but because this gene lies far outside the complex *FCGR2/3* locus and no disease associations have been described yet, these SNPs are beyond the scope of this review.

We provide an overview of the currently known genetic variation in low-to-medium-affinity FcγRs, with a focus on the genetic challenges in characterizing this locus, nomenclature of the variations, functional consequences, disease associations with specific diseases and in general, and will discuss the potential of *FCGR2/3* genotyping for personalized medicine.

## Low-to-Medium-Affinity Fc-Gamma Receptors

IgG-FcγR interactions depend on the IgG subclass (IgG1, IgG2, IgG3, and IgG4) and IgG-Fc glycosylation structure of p.Asn297 in the IgG protein, as well as on the specific FcγR and variation within its amino acid sequence by genetic polymorphisms (7, 8). A schematic representation of the low-to-medium-affinity Fc-gamma receptors and the approximate location of the genetic variants is provided in Figure 1.

FcγRIIA (CD32a) consists of a single polypeptide chain which contains an immunoreceptor tyrosine-based activating motif (ITAM) in the intracellular domain. FcγRIIA is the most widely expressed isoform of FcγRII and is found on monocytes, macrophages, dendritic cells, neutrophils, and platelets. It can induce many different cellular defense mechanisms such as phagocytosis of IgG-opsonized targets, antibody-dependent cellular cytotoxicity (ADCC), production of reactive oxygen species (ROS), and cytokine production.

FcγRIIB (CD32b) is the only FcγR that results in an inhibitory signal to the cell, which is transferred by the immunoreceptor tyrosine-based inhibitory motif (ITIM) on its intracellular signaling domain. FcγRIIB is found in two isoforms deriving from two different transcripts (Figure 1), FcγRIIB-1 and FcγRIIB-2, with FcγRIIB-1 having an additional intracellular exon in between the transmembrane and signaling domains. FcγRIIB-1 is highly expressed on B cells, where it constitutes the only surface-expressed FcγR, and co-crosslinking of FcγRIIB-1 with the B cell receptor (BCR) inhibits activating signals induced by the BCR. Other cell types also express FcγRIIB, albeit at much lower levels, and on these cells FcγRIIB-2 is the main transcript expressed. These cells include a subset of monocytes, macrophages, and dendritic cells. Expression of FcγRIIB can also be detected on neutrophils and NK cells, but only in individuals with certain genotypes (9–11). When transfected in COS-1 cells, FcγRIIB can inhibit pro-phagocytic signals induced by activating FcγRs, balancing the immune response against IgG-opsonized targets (12), but it remains currently unknown if this mechanism is also involved in myeloid cells. Interestingly, at phagocytic cups, FcγRIIB may be relatively excluded whereas FcγRIIA is enriched, likely due to their difference in IgG affinity, which may affect the ability of FcγRIIB to exert inhibitory signals (13).

FcγRIIC (CD32c) has long been considered not to be expressed at all, as its gene (*FCGR2C*) was thought to be a pseudogene (14, 15), and therefore relatively little was known about the expression pattern of this receptor. In 1998, FcγRIIC was first found on NK cells of individuals that carry an open reading frame (ORF) of this receptor (p.57Gln, *FCGR2C*-ORF), as opposed to the majority of individuals in which this receptor is indeed a pseudogene and cannot be expressed as a result of a stop codon in exon3 (p.57Ter, *FCGR2C*-Stop) (16). Determining the cellular expression pattern of FcγRIIC has long been complicated because the extracellular domains are identical to FcγRIIB, but specific detection of FcγRIIC is possible by comparison of cellular expression between individuals that can or cannot express FcγRIIC as a result of the stop codon, detection of FCGR2C mRNA and western blots of immunoprecipitated FcγRIIC. We now know that FcγRIIC can be expressed on NK cells, neutrophils, monocytes (9), and macrophages (17). This receptor has also been reported to be expressed on B cells (18) although expression on B cells could not be reproduced in our own laboratory (17). Obviously, FcγRIIC can only be functional in individuals with an *FCGR2C*-ORF. Although expression on NK cells is relatively low, it has been shown to be capable of inducing killing of target cells in a redirected ADCC assay (19), functioning as an activating receptor.

FcγRIIIA (CD16a) has two extracellular (EC) Ig-like domains, involved in binding of IgG, a transmembrane (TM) domain and a short intracellular (IC) domain. The TM domain associates with adaptor proteins containing an immunoreceptor tyrosine-based activating motif (ITAM) to induce intracellular signaling. In monocytes and macrophages, this receptor associates with the FcRγ-chain, while in NK cells it associates with the CD3 ζ-chain (20–22). Association with these adaptor proteins is not only essential for signaling and maintaining stable expression, but also for targeting the receptor to the cell membrane (22). FcγRIIIA expressed on NK cells can induce ADCC by these cells (23), and on phagocytes it can induce phagocytosis (24). Recently, it was suggested that FcγRIIIA is also expressed in low levels on neutrophils (25), which is surprising since it was never found before and contradicts the finding that two donors completely deficient for FcγRIIIB did not show any staining on neutrophils with a MoAb (3G8) that recognizes both FcγRIIIA and FcγRIIIB (11).

FcγRIIIB (CD16b) is a GPI-anchored protein, expressed in high numbers on neutrophils, and sometimes on eosinophils. As it does not have a transmembrane domain, it cannot associate with FcRγ or the ζ-chain. FcγRIIIB is not capable of IgG-induced production of ROS (26). However, it does contribute in *in vitro* experiments to the exocytosis of neutrophil granule proteins (27) and Ca^2+^ influx (28), and may also cooperate with FcγRIIA on the same neutrophil to induce such responses (29). Because FcγRIIIB can induce these responses, FcγRIIIB has often been classified as an activating receptor, although the exact mechanism(s) by which FcγRIIIB activates cells are still unclear (30, 31). Nowadays FcγRIIIB is mainly seen as a decoy receptor (32, 33) as it has a clear role in binding to—and mediating internalization of—soluble immune complexes in neutrophils (34, 35). In fact, FcγRIIIB can also decrease antibody-mediated trogocytosis by neutrophils (36). In FcγR-deficient mice transgenic for human FcyRIIIB, FcyRIIIB mediates the interaction of neutrophils with soluble immune complexes and render the neutrophil susceptible to tissue adhesion and capillary transmigration (37). FcyRIIIB also allows flowing neutrophils to tether to IgG and immune complexes (38).

## The *FCGR2/3* Locus

**Figure 2:**
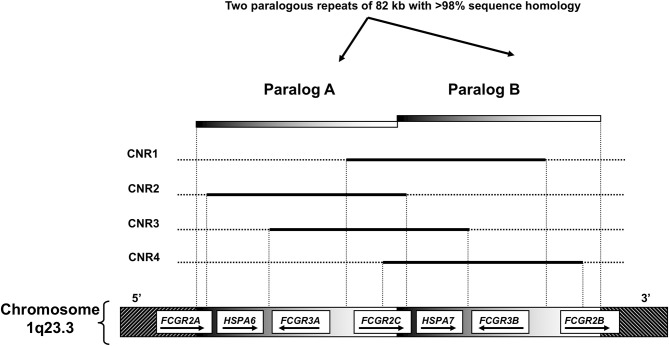
Overview of the *FCGR2/3* locus. A Structural overview of the locus, with the orientation of the genes indicated by arrows. B Overview of copy number variation at the locus. Four combinations (copy number variable regions, CNRs) of FcγR genes have been shown to occur in duplication/deletion. Black lines indicate which genes are involved in copy number variation (CNV).

**Figure 3:**
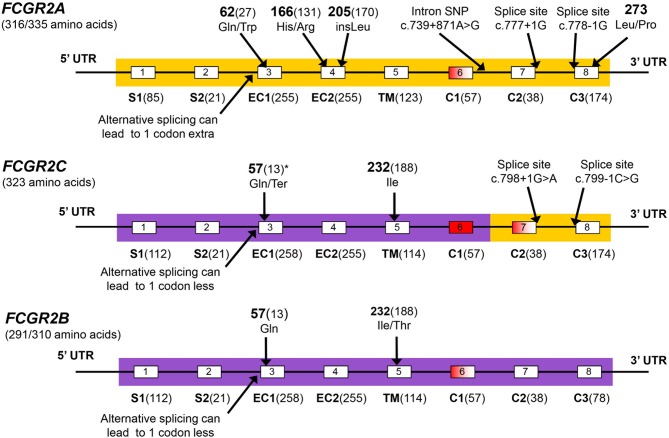
*FCGR2* exons. The *FCGR2C* gene is the crossover product from an unequal crossover between *FCGR2A* and *FCGR2B*. Coloring of *FCGR2C* matches the color of the other *FCGR2* genes in the parts where it is highly homologous to that gene. Exons are shown by boxes, white exons are included in all transcripts, red exons are always spliced out, red-shaded exons are spliced out in some transcripts but retained in others. Exon names are below and followed by the number of coding base pairs in that exon. S1, S2, signal peptides; EC1, EC2, extracellular domains; TM, transmembrane domain; C1, C2, C3, cytoplasmic domains. The C3 exons contain an immunoreceptor tyrosine-based activation motif (ITAM) in *FCGR2A* and *FCGR2C*, and contains an immunoreceptor tyrosine-based inhibitory motif (ITIM) in *FCGR2B*. There is a potential confusion with regard to exon numbering in *FCGR2* genes: In the *FCGR2B* gene, transcripts exist that do (FCGR2B1) or do not (FCGR2B2) retain the 57 bp exon6, dependent on the cell type in which the receptor is expressed. In *FCGR2A* and *FCGR2C* a very homologous exon6 is present on genomic level, but this exon is always spliced out in FCGR2A and FCGR2C transcripts. Only in some rare cases exon6 is retained in FCGR2A, which results in a gain-of-function FcγRIIA isoform (39). The final two exons of *FCGR2A* and -*2C* are often designated exon6 and 7, but to reflect the homology between the 3 *FCGR2* genes, we chose to include the potential exon6 in the nomenclature of all *FCGR2* genes, designating the final 2 exons exon7 and 8. However, we do not include the base pairs of this exon6 when indicating the nucleotide positions in the FCGR2A and FCGR2C transcripts, because this is not normally done in the literature. In FCGR2A transcripts, further inconsistencies exist as a result of alternative splicing at the beginning of exon3 because of two adjacent splice acceptor sites that can both be used. The most commonly used nucleotide and amino acid numbering is derived from the shorter transcript in which the 3' splice acceptor site is used, so we chose to use this transcript for nucleotide numbering throughout this thesis. Several SNPs are indicated in the figure, all are indicated by their amino-acid position in the full protein, followed by the amino acid position excluding signal peptides between brackets for some of the SNP which are commonly known by that position. ^*^The p.Gln57Ter SNP in *FCGR2C* is part of a haplotype of 8 SNPs in intron2 and exon3 in *FCGR2C*, this whole haplotype is identical to *FCGR2B* in the case of p.57Gln (40). p57Gln is the only non-synonymous coding SNP in this haplotype.

**Figure 4:**
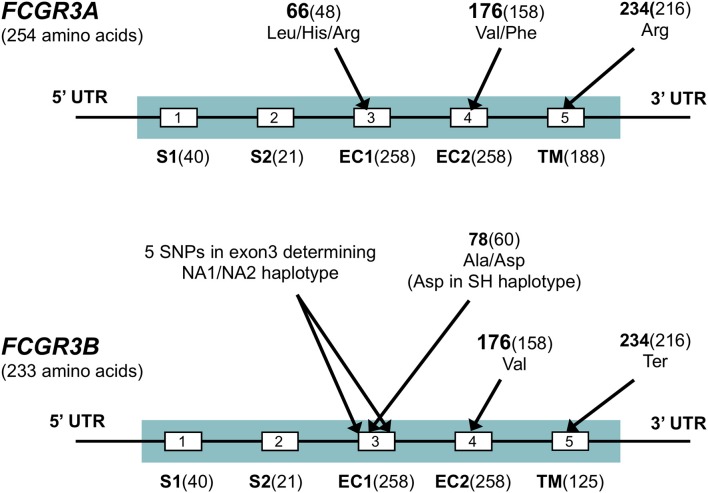
*FCGR3* exons. Exons are shown by boxes. Exon names are below and followed by the number of coding base pairs in that exon. S1, S2, signal peptides; EC1, EC2, extracellular domains; TM: transmembrane domain. Several SNPs are indicated in the figure, all are indicated by their amino-acid position in the full protein, followed by the amino acid position excluding signal peptides between brackets for some of the SNP which are commonly known by that position. Sequences for *FCGR3A* and *FCGR3B* are very similar, but four non-synonymous differences in the coding sequence exist, most notably the stop codon at p.234 in *FCGR3B* as indicated in the figure, which truncates the transmembrane domain of this receptor. Other amino acid differences between *FCGR3A* and *FCGR3B* include p.147 (Gly in *FCGR3A*, Asp in *FCGR3B*), p.158 (Tyr in *FCGR3A*, His in *FCGR3B*) and p.203 (Phe in *FCGR3A*, Ser in *FCGR3B*) (41). A set of 6 SNPs in exon3 of *FCGR3B* form three rather well-defined haplotypes, the *FCGR3B*-NA1, -NA2 and -SH. *FCGR3A* is identical to NA1 at some sites, but to NA2 at others (41).

The low-to-medium-affinity FcγRs; FcγRIIA, FcγRIIB, FcγRIIC, FcγRIIIA and FcγRIIIB are encoded respectively by *FCGR2A, FCGR2B, FCGR2C, FCGR3A*, and *FCGR3B*. All these genes are located in a cluster at 1q23.3 in the *FCGR2/3* locus (Figure 2). The locus consists of two 82 kb paralogous repeats with >98% sequence homology, that were formed as the result of an unequal crossover event (15). This unequal crossover event between *FCGR2A* and *FCGR2B*, the two genes that flank the region, has led to a segmental duplication in which *FCGR2C* was formed (15), with the resulting *FCGR2C* gene being highly homologous to *FCGR2B* in the first six exons and highly homologous to *FCGR2A* in the last 2 exons. Figure 3 provides an overview of the differences between the three *FCGR2* genes. Furthermore, the segmental duplication created the two different *FCGR3* genes, *FCGR3A* and *FCGR3B*, which are also highly homologous in sequence (Figure 4). The genes encoding the classic FcγRs are highly polymorphic and functionally relevant genetic variations have been described for all low-to-medium-affinity FcγRs. An overview of the functionally relevant SNPs, is given in Table 1, and the approximate locations within the FcγRs are shown in Figure 1. The functional consequences of the SNPs are discussed below.

**Table 1:** Overview of single nucleotide polymorphisms (SNPs) and copy number variation (CNV) at the *FCGR2/3* locus.

**Rs #**	Nucleotide^**^	Amino acid position^***^	**Amino acid**	**Functional change**	**Associations with disease**
***FCGR2A***					
rs201218628	c.184C c.185A	**62** (27)	Gln	Possibly reduced signaling (42), no influence on expression (43)	
	c.184T c.185G		Trp		
rs1801274	c.497A	**166** (131)	His	higher affinity for human IgG (44)	**KD** **(****45****)**, **childhood ITP** **(****46**, **47****)**, possibly Guillain Barre Syndrome (48, 49)
	c.497G		Arg		**SLE** **(****50**, **51****)**, meningococcal sepsis (52), sepsis (53, 54)
rs150311303	c.612_613 insCTT	**205** (170)	Leu	higher affinity for human IgG (55)	
rs72717038	c.739 +871 A>G	–	–	G retains exon 6, increased signaling (39)	Anaphylaxis in patients with hypogammaglobulinemia (39)
rs382627	c.818C	**273**	Leu		
	c.818T		Pro	Introduced by deletion of CNR2, decreased expression (56)	
***FCGR2B***					
rs143796418	−386 C>G^***^	–	–	Promoter haplotypes 2B.1, 2B.2 and 2B.4 influences expression (57, 58)	2B.4 haplotype associated with susceptibility to SLE (57, 58)
rs780467580	−120 T>A^***^	–	–		
rs755222686		**106**	Asn		
	c.316_318del		Del	Deletion abolishes IgG-binding	Increased serum levels of IgG1 and IgG3 (59)
rs1050501	c.695T	**232**	Ile		
	c.695C		Thr	Excludes receptor from lipid rafts (60, 61)	**Susceptibility to SLE** **(****50**, **60****)**, protection against malaria (62)
***FCGR2C***					
CNV	–	–	–	Expression levels (only in *FCGR2C*-ORF) (63)	KD (unexplained mechanism) (64)
rs149754834	−386 C>G^***^	–	–	Promoter haplotypes 2B.1, 2B.2 (possibly 2B.4) functional change unknown	
rs34701572	−120 T>A^***^	–	–		
rs759550223	c.169T	**57** (13)	Ter	Stop codon, no expression of FcγRIIC	
	c.169C		Gln	Results in an open reading frame (ORF) and expression of FcγRIIC (9, 19)	ITP (19, 65), Kawasaki Disease (43), IgG subclass deficiency (66)
rs114945036	c.134-96C>T	–		unknown	Minor allele associated with HIV disease progression (67)
rs138747765	c.353C>T	**118**	Ile/Thr	unknown	Possibly linked to rs114945036
rs76277413	c.798 +1 A>G	–	–	A causes exon7 to be spliced out (9)	
rs430178	c.799−1 C>G	–	–	C leads to retention of 62 intronic base pairs (9)	
***FCGR3A***					
CNV	–	–	–	Expression levels Decreased ADCC (1 copy vs. 2 copies)	SLE (both <2 and >2 copies) (68), anti-GBM disease (>2 copies) (69)
rs10127939	c.197T	**66** (48)	Leu	Linked to rs396991 (70)	
	c.197A		Arg	Increased IgG binding in presence of *FCGR3A*-p.176Val (71)	
	c.197G		His	Increased IgG binding in presence of *FCGR3A*-p.176Val (71)May result in functional defects in NK cells (72, 73)	Homozygosity associated with severe Herpes infections (72–74)
rs396991	c.526G	**176** (158)	Val	higher affinity for human IgG (44, 70)	Susceptibility to ITP (19, 47, 75), RA (76) and ulcerative colitis (77)
	c.526T		Phe		**Susceptibility to SLE** **(****50****)**
***FCGR3B***					
CNV	–	–	–	Expression levels Uptake of immune complexes	**SLE (<2 copies)** **(****78****), Sjögren syndrome (<2 copies)** **(****79****), systemic sclerosis (<2 copies)** **(****80****)**, **RA (<2 copies)** **(****78**, **81**, **82****)**, Ulcerative Colitis (<2 copies) (83), Ankylosing Spondylitis (84), ANCA-associated vasculitis (<2 copies) (85) Bullous Pemphigoid (inverse relation with CNV) (86)
rs200688856	c.108G	**36**	Arg	NA1^****^	
	c.108C		Ser	NA2 and SH^****^	**NA2 associated with susceptibility to SLE** **(****50****)**
rs527909462	c.114C	**38**	Leu	NA1	
	c.114T		Leu	NA2 and SH	
rs448740	c.194A	**65**	Asn	NA1	
	c.194G		Ser	NA2 and SH	
rs5030738	c.233C	**78**	Ala	NA1 and NA2	
	c.233A		Asp	SH	
rs147574249	c.244G	**82**	Asp	NA1	
	c.244A		Asn	NA2 and SH	
rs2290834	c.316G	**106**	Val	NA1	
	c.316A		Ile	NA2 and SH	

*Associations found in meta-analyses or GWAS studies are indicated in bold*.

*For each SNP, Rs numbers (Reference SNP cluster ID, the common identification method of SNPs as included in the dbSNP database), nucleotide and amino acid positions, functional changes, and associations with disease are shown*.

* *Nucleotide numbering excludes exon6 in FCGR2A and FCGR2C transcripts, because this exon is spliced out from these transcripts, but includes exon 6 in FCGR2B, in which it is retained in many transcripts (splice variant known as FCGR2B1)*.

*In FCGR2A transcripts, inconsistencies exist as a result of alternative splicing at the beginning of exon3 because of two adjacent splice acceptor sites that can both be used. The most commonly used amino-acid numbering is derived from the shorter transcript in which the 3′ splice acceptor site is used, so we chose to use this transcript for nucleotide numbering throughout this manuscript*.

** *Inconsistencies exist in the amino-acid numbering used in the literature, because some SNPs are named by the position when including the signal peptides, and others are named by their position in the mature protein, excluding the signal peptides. To comply with the official HGVS guidelines, we propose to use the amino acid in the full protein and have done this throughout the manuscript. In this table, position in the mature protein is shown between brackets for some of the SNP which are commonly known by that position*.

*** *Relative to the start of translation. Three haplotypes have been described: 2B.1 (−386, −120T); 2B.2 (−386C, −120T) and 2B.4 (−386C, −120A). −386G, −120A has never been found to date*.

**** The set of 6 SNPs in FCGR3B determines the NA1,NA2 and SH haplotypes. These are the three major haplotypes that exist, although rare additional variants have been reported (87). The term “NA” is derived from “Neutrophil Antigen.” The term “SH” derives from the fact that an alloantibody recognizing this antigen was first found in serum “h” among several different investigated sera (Jürgen Bux, personal communication). FCGR3B-NA1 and -NA2 nucleotide sequences differ at five positions (c.108G>C, c.114C>T, c.194A>G, c.244G>A and c.316G>A), with four predicted amino acid differences (p.Arg36Ser, p.Asn65Ser, p.Asp82Asn and p.Val106Ile for NA1 and NA2, respectively). As a consequence, the NA2 variant has two additional N-linked glycosylation sites compared to NA1 (the p.65Ser of NA2 completes a consensus sequence for N-linked glycosylation with the non-polymorphic p.63Asn residue, and the p.82Asn of NA2 forms a consensus sequence with the non-polymorphic p.84Ser) (88). The SH variant is identical to NA2 at the five positions that distinguish NA1 from NA2, but differs from both variants at one additional position (c.233C>A), resulting in an p.Ala78Asp amino acid change that predicts a change in the tertiary structure of the protein (89). Additional complexity is added by the discovery of rare individuals carrying other mutations within this gene or different combinations of these nucleotide polymorphisms (87, 90), indicating that the NA1/NA2/SH typing is incomplete. Sometimes, the NA1/NA2/SH haplotypes are indicated, respectively, as FCGR3B^*^01, FCGR3B^*^02 and FCGR3B^*^03, to prevent confusion with the nomenclature for antigenic epitopes determined by these haplotypes. These haplotypes determine the allotypic variants of the Human Neutrophil Antigen1 (HNA1), which is involved in allo-immunization against neutrophilic granulocytes. The HNA classification system recognizes HNA1a (encoded by FCGR3B-NA1), HNA1b (encoded by FCGR3B-NA2 and FCGR3B-SH) and HNA1c (encoded by FCGR3B-SH) (41, 89, 91–93). Recently, a fourth antigenic epitope was described (HNA1d, also encoded by FCGR3B-NA2) (93).

*Note that the nucleotide positions as indicated here are indicating the position in the coding sequence, which differs from nucleotide positions often used in the literature for these haplotypes, as derived from Ravetch and Perussia (94) who used a nucleotide numbering not related to the coding sequence of FCGR3B, which includes 33 additional nucleotides of the 5′UTR*.

Besides being polymorphic, some of the low-to-medium-affinity *FCGR* genes are subject to gene copy number variation (CNV). Although several large-scale studies on CNV have suggested that human *FCGR2A* and *FCGR2B* are candidate genes for CNV (95, 96), our group has shown previously that this is not the case. In fact, CNV in the *FCGR* locus is restricted to *FCGR2C, FCGR3A*, and *FCGR3B* (63). CNV at the *FCGR2/3* locus always occurs in distinct copy number regions (CNRs) that consist of a complete stretch of 82 kb generated by non-allelic homologous recombination (NAHR) (Figure 2) (56, 97, 98). The clear distinction in CNRs suggests that there must be hotspots for NAHR breakpoints. Breakpoints for the most common CNR1 have been studied in more detail and it appears that these consist of several different breakpoints (81, 97). Exact localization of breakpoints for CNR1 may not always be possible because many potential breakpoints for CNR1 lie within a 24.5 kb block in which no genuine paralogous sequence variants (PSVs) are present (i.e., no clear distinction between paralog A and paralog B can be made and therefore no absolute conclusion on the position of a breakpoint) (99). This block comprises of both the 3′ end of the intergenic region between *FCGR3B* and *FCGR2B* and the first exons of *FCGR2B*. Breakpoints for the rare CNR4 (which have a breakpoint distal of exon3 of *FCGR2B*) can also lie within this 24.5 kb block. This 24.5 kb “block” may however be a result of a combination of different (smaller) gene conversion events, and Rahbari et al. later showed that it was possible to define breakpoints even within this 24.5 kb block (81).

### Nomenclature of Variations at the *FCGR2/3* Locus

Many inconsistencies in the nomenclature of SNPs at the *FCGR2/3* locus exist, because some SNPs are commonly indicated by the amino acid position in the mature protein (from which the signal peptides have been cleaved off), whereas others are indicated by the amino acid position in the full protein. For some SNPs, both positions are used in the literature, which leads to confusion. We propose to use the amino acid positions in the full protein to avoid possible misunderstanding, following the guidelines of the Human Genome Variation Society (HGVS) nomenclature (100) and have used these positions throughout this review. Table 1 lists also the frequently used positions in the mature protein for some of the SNPs. Further confusion can derive from alternative exon numbering in *FCGR2* genes, as explained in Figure 3.

### Genetic Analysis of the *FCGR2/3* Locus

As a result of the high sequence homology between the genes, genotyping of this locus is very complicated, and it is important to realize that commonly used genome databases such as Ensembl or NCBI BLAST are not in all cases accurate in the distinction between a SNP in one of the *FCGR* genes and a genuine difference between two homologous *FCGR* genes (PSV). Detailed knowledge of the organization of the locus and adequate primer design that enables distinction between the paralogs is essential for a proper genetic analysis, and recommendations for analyzing this complex locus have been published before (101). A good source for distinguishing SNPs from PSVs is provided in a Supplementary Table in the article by Mueller et al. (99).

Copy number variation at the locus is commonly determined by multiplex ligation-dependent probe amplification (MLPA) or by paralogue ratio test (PRT) which were concordant in most but not all cases (102). Recently, a method was described that uses data from whole-genome array comparative genomic hybridization (aCGH) to distinguish heterozygous deletion alleles that either include *FCGR3A* (i.e., CNR2 and CNR3) or include *FCGR3B* (i.e., CNR1 and CNR4) (81), which allows for a more high throughput method to determine CNV at the *FCGR2/3* locus, although only heterozygous deletion alleles could be called with reasonable accuracy. An attempt has also been made to determine *FCGR3A* and *FCGR3B* CNV from intensity values derived from an Immunochip platform, allowing the authors to determine CNV in >18,000 individuals (103). This was said to reliably identify cases with 0,1,2 or more copies, although 3 copies could not be reliably be distinguished from higher copy numbers. The authors did however not validate their findings with standard techniques to determine CNV at the *FCGR2/3* locus, such as MLPA or the paralogue ratio test (PRT) (104). In our opinion, the fact that in this study the authors could not find any relation of *FCGR3B* CNV with expression of FcγRIIIB on neutrophils puts serious doubts on the reliability of these data, as expression of FcγRIIIB clearly correlates with CNV of *FCGR3B* as measured by PRT (105) and MLPA (11, 43, 86).

Similarly, next generation sequencing techniques are currently insufficient to determine *FCGCR2/3* SNPs, as multiple variants were mistyped in subjects that were genotyped by whole-exome sequencing (56). Thus, it appears that next generation sequencing techniques will have to be improved greatly before such methods can be used to adequately genotype the *FCGR2/3* locus (as well as other complicated loci with duplications and high homology), and it is not sure whether such methods will be present for high-throughput analysis in the near future.

## Functional Consequences of SNPs in the *FCGR2/3* Genes

In *FCGR2A*, encoding for FcγRIIA, a well-known SNP rs1801724 is present which results in either a histidine or an arginine at position 166 in the full protein: p.His166Arg (formerly known as p.His131Arg). p.His166Arg is in the IgG binding domain (EC2) (106); FcγRIIA-p.166His has a higher binding affinity for IgG1 and especially IgG2, as compared to FcγRIIA-p.166Arg, but binding to IgG3 and IgG4 is similar for both variants (44). Functionally, mononuclear cells from homozygous FcγRIIA-p.166His individuals produce more IL-1beta when stimulated with IgG2 than heterozygous and homozygous FcγRIIA-p.166Arg individuals (107). Similarly, neutrophils from homozygous FcγRIIA-p.166His individuals have been shown to have increased phagocytosis and degranulation in response to serum-opsonized bacteria and increased rosette formation and phagocytosis in presence of IgG3 anti-RhD sensitized erythrocytes when compared to homozygous FcγRIIA-p.166Arg individuals (108, 109).

In addition to the well-studied *FCGR2A*-p.His166Arg, several other functional SNPs have been described in *FCGR2A*: *FCGR2A*-p.Gln62Trp (formerly known as p.Gln27Trp), a combined SNP of two adjacent nucleotides (known separately as rs9427397 and rs 9427398, and combined as rs201218628) is in linkage disequilibrium with *FCGR2C-*ORF and the *FCGR2B* promoter haplotype 2B.4 (43). Compared to p.62Gln, the p.62Trp allele shows similar FcγRII expression amongst neutrophils and monocytes and, even though slightly reduced calcium signaling has been observed in overexpressed cell lines, does not affect neutrophil ADCC *in vitro* (42). Because of the linkage disequilibrium and given these functional data, it is more likely that 2B.4 variant or *FCGR2C*-ORF confer the increased risk for autoimmune disease than *FCGR2A*-p.62Trp.

At position p.273, which normally is a Leucine in *FCGR2A*, a Proline can be introduced into *FCGR2A* by deletion of CNR2 which causes the fusion of the proximal part of *FCGR2A* and the distal part of *FCGR2C* causing a chimeric *FCGR2A/2C* gene (56). The Leu/Pro difference is the only amino acid different in this region of *FCGR2A* and *FCGR2C*, although additional variants exist in the 3'UTR (56). The chimeric *FCGR2A/2C* gene shows lower expression levels and lower generation of reactive oxygen species in comparison with the wild-type *FCGR2A*. This variant could either be seen as a SNP or as the result of the fusion of two genes. *FCGR2A*-P.Leu273Pro has also been described as a SNP (rs382627, currently flagged as suspect in dbSNP) in a Japanese individual, although it has not been tested whether this individual also had a deletion of CNR2 (110). Compared to leucine, the proline variant was shown in this report to have an increased signaling capacity when expressed in cell lines (110).

Another SNP that may influence the function of FcγRIIA is the intronic rs72717038, which could cause retention of exon6 which is associated with increased signaling capacity (39, 111).

Finally, *FCGR2A*-p.Val204_Gln205insLeu (rs150311303) is a SNP observed with a minor allele frequency of 8.3% in an African population and confers a higher affinity for human IgG (55).

**Figure 5:**
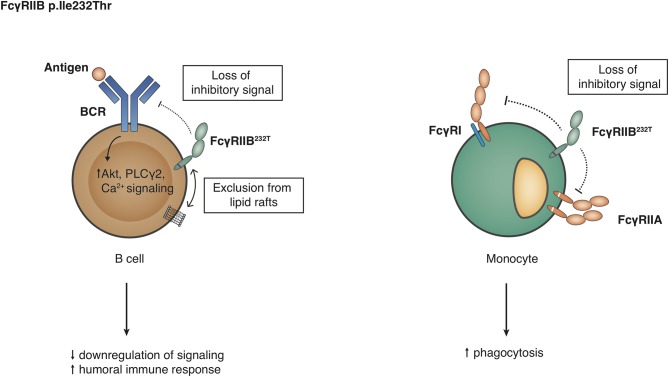
Functional consequences of the *FCGR2B-*p.Ile232Thr variant. Detailed explanations are given in the main text.

*FCGR2B*, encoding for FcγRIIB, also exists in two allelic variants, containing either an isoleucine or a threonine at position 232 in the TM domain (112). As this SNP (p.Ile232Thr) does not affect the IgG-binding EC domains, it has no influence on the binding affinity. However, its localization at the TM domain results in differences in downstream signaling and subsequent inhibition of FcγRI signaling in macrophages and BCR signaling in B cells. In particular, p.Ile232 provides stronger inhibitory signaling than p.Thr232, and this is caused by the exclusion from lipid rafts of FcγRIIB-p.Thr232 (60, 61) (Figure 5). Dendritic cells (DC) also express FcγRIIB, affecting DC maturation end T cell stimulation (113–115), hence FcγRIIB-p.Thr232 may also influence the function of these cells. Other genetic variations influence the *expression* of FcγRIIB. For instance, in individuals with a CNR1 deletion in the *FCGR* locus, FcγRIIB can surprisingly also be expressed on the surface of NK cells (9, 11, 99). Expression of FcγRIIB in neutrophils, monocytes or B cells is hardly affected by this deletion (9). Furthermore, two SNPs in the promoter of *FCGR2B* and *FCGR2C*, a guanine or cytosine at position −386 and a thymine or adenine at position −120 relative to the start codon, form four haplotypes of which one (−386G, −120A; 2B.3) has never been found in any individual thus far. In case of *FCGR2B*, the rare 2B.4 promoter haplotype (−386C, −120A) appeared to have higher transcriptional activity than the wild-type promoter 2B.1 (−386G, −120T) (57), resulting in increased expression on neutrophils (10, 11), monocytes (11) whereas the result of this 2B.4 promoter on the expression of FcγRIIB on B cells is less clear and conclusions from various reports range from increased expression (57), no effect (11) to a *decreased* expression of FcγRIIB on B cells (58); which may differ among different B cell subsets. Functionally, 2B.4 led to a stronger inhibition of B cell receptor signaling without affecting surface expression levels as such (116).

Recently, a rare in-frame deletion c.316_318del, p.Asn106del, rs755222686, was described in the Icelandic population that abolishes IgG binding to FcγRIIb (59). The asparagine residue at position 106 is part of an N-linked glycosylation site, but the absent binding of IgG was not a result of the removal of the glycan, because the same glycan was found at the adjacent asparagine at position 105 in protein encoded by the deletion allele. p.Asn106del was associated with increased levels of IgG1 and IgG3.

In *FCGR2C*, the previously mentioned p.Gln57Ter SNP (sometimes known as p.Gln13Ter determines whether or not individuals can express FcγRIIC at all. This mutation results in either an open reading frame (classic *FCGR2C*-ORF, allele frequency ~10–15% in Caucasians) or a stop codon (*FCGR2C*-Stop) (19). Classically, ORF/Stop genotyping of individuals is done based on this SNP alone. However, we have recently found that some individuals carry splice site mutations in intron7, which leads to alternative transcripts, causing a frameshift in exon8 and the introduction of novel stop codons, leading to an almost complete loss of FcγRIIC expression (9, 43). Genotyping of *FCGR2C* should therefore include these novel mutations to provide an accurate prediction for FcγRIIC expression.

**Figure 6:**
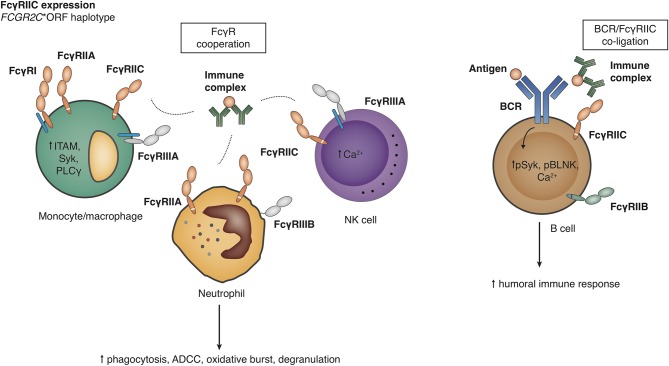
Functional consequences of the *FCGR2C-*open reading frame (ORF) haplotype. Detailed explanations are given in the main text. Right panel, Effects on the level of B cells have been described by Li et al. (18), but other analyses have not shown FcγRIIC expression on B cell surface in *FCGR2C-*ORF individuals (17).

FcyRIIC on NK cells offers an antibody-mediated, FcyRIIIA-independent pathway to trigger Ca^2+^ signaling and ADCC (16, 117) (Figure 6). Because of the identity of the intracellular signaling elements of FcyRIIC and FcyRIIA, it can be speculated that FcyRIIC acts as an independent phagocytic receptor on myeloid cells, but this has not been shown experimentally. Transgenic mice expressing FcyRIIC in B cells show enhanced humoral immune responses after T cell dependent and T-independent vaccination (18). Altogether, classic *FCGR2C*-ORF may thus predispose to autoimmune disease either by providing attenuated innate immune responses or by enhancing the humoral immune response.

In *FCGR2C*, the promoter haplotypes of *FCGR2B* as mentioned above, can also be found. In general, only the wildtype and one other promoter haplotype (−386C, −120T; 2B.2) are found. The 2B.2 haplotype is linked to p.57Gln (19, 43).

Recently, a haplotype of several SNPs in and around exon3 has gained a lot of attention because of a possible association with HIV vaccine efficacy. This haplotype consists of c.134-96C>T (rs114945036), p.(Thr118Ile) (rs138747765) and c.391+111G>A (rs78603008) and was associated with vaccine efficacy despite the fact that all but one study participants had a p.57Ter allele. The function of the SNPs in the haplotype is unknown. However, the results of this study must be taken with great caution because the primers used to distinguish *FCGR2C* from *FCGR2B* have used specific sites that, according to Mueller et al. (99), are all polymorphic in *FCGR2C* and can completely resemble *FCGR2B*. The authors may therefore have missed some *FCGR2C* alleles in their analysis which may have skewed the results. Later, the haplotype has been reanalyzed in a South African cohort (118) using a specific long range PCR to sequence *FCGR2C*; these authors have indeed found the c.134-96C>T SNP but not the other SNPs of the haplotype in their cohort. The minor allele of the c.134-96C>T SNP was later found by the same group to be associated with increased odds of HIV-1 disease progression (67).

The FcγRIIIA-encoding *FCGR3A* gene contains a SNP that results in either a valine or a phenylalanine at position 176 (p.Val176Phe), formerly known as p.Val158Phe, located in the EC2 domain (119). FcγRIIIA-p.176Val has a higher binding affinity for all human IgG classes compared to FcγRIIIA-p.176Phe (44). In ADCC assays, NK cells from FcγRIIIA-p.176Val donors show increased killing of target cells that are opsonized with sub-saturating levels of the human anti-CD20 MoAb Rituximab (23). Another SNP in the *FCGR3A* gene is a triallelic SNP at position 66; p.66Leu/Arg/His, also formerly known as p.48Leu/Arg/His which is located in the EC1 domain which is not directly involved in binding IgG. Rare homozygosity of p.66His was first described in a patient with recurrent Herpes infections (72) and was later found in two other patients with decreased clearance of Herpes infections (73, 74) and suggested to be a congenital immunodeficiency (73). However, homozygosity for p.66His was also found in a cohort of healthy individuals of European descent (genotype frequency 0.6%) and African descent (genotype frequency 0.1%) (71). Apparently, the clinical phenotype of homozygity for p.66His differs between individuals and recurrent Herpes infections may be associated with but are not directly caused by the mutation.

The FcγRIIIB-encoding *FCGR3B* gene exists in three polymorphic variant proteins, best known as the NA1, NA2, and SH haplotypes. These haplotypes consist of a set of 6 SNPs in exon3 of *FCGR3B* (Table 1 and Figure 4). The *FCGR3B* variants encoded by these haplotypes determine the allotypic variants of the Human Neutrophil Antigen1 (HNA1) (91), which is involved in allo-immunization against neutrophilic granulocytes. The NA1, NA2 and SH haplotypes are sometimes referred to in the literature as HNA1a, HNA1b and HNA1c, respectively, although the latter nomenclature in strict sense determines antigenic epitopes and not genetic haplotypes (see Table 1 for a detailed description). Apart from determining allo-immunization against neutrophils, these haplotypes are known to have functional differences. Compared to NA1, the NA2 and SH variants have two additional N-linked glycosylation sites. The SH variant differs from NA1 and NA2 by a p.Ala78Asp amino acid change that predicts a change in the tertiary structure of the protein (89), although the actual functional consequences of this SNP are not well-characterized. While the binding affinities for IgG1 and IgG3 appear similar between NA1, NA2 and SH (44), neutrophils from FcγRIIIB-NA1NA1 individuals bind and phagocytize IgG-opsonized bacteria and red blood cells more efficiently than those from FcγRIIIB-NA1NA2 and -NA2NA2 individuals (108, 120). It is not known whether the SH is functionally different from the otherwise similar NA2 variant.

## Functional Consequences of CNV in the *FCGR2/3* Genes

**Figure 7:**
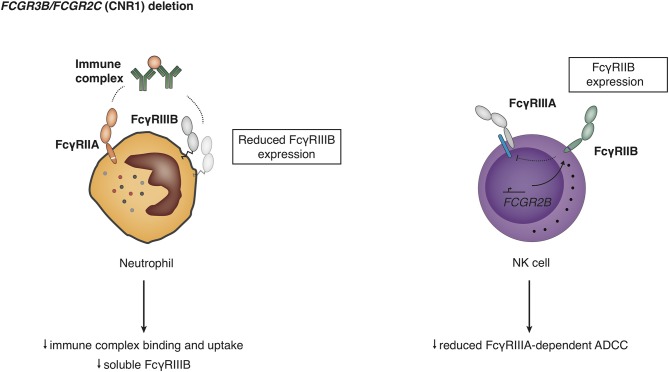
Functional consequences of deletion in the copy number region 1 (CNR1). Detailed explanations are given in the main text.

CNV (Figure 2) results in differences in expression levels of FcγRIIIB, where an increase in gene copies of *FCGR3B* very clearly leads to a higher receptor expression at the cell surface (11, 63, 105, 121, 122) and higher mRNA levels (43, 86), and decreased copy number associated with lower soluble serum FcyRIIIB levels released from activated neutrophils (123) (Figure 7). Functionally, increased expression of FcγRIIIB leads to higher binding and uptake of immune complexes by neutrophils (105) and may be associated with increased ROS production (86), which is an intriguing finding because FcγRIIIB has been shown not to contribute to ROS production (27). Interestingly, individuals who are homozygous for a CNR1 deletion, i.e., have no copies of *FCGR3B*, are generally healthy and apparently not at risk for overwhelming bacterial infections, suggesting that their neutrophil function is sufficient to maintain immune homeostasis (122).

In addition, a CNR1 deletion leads to expression of FcγRIIB on NK cells (9, 99), which is presumed to result from the molecular effects of the promoter rearrangement, and the ectopic expression of FcγRIIB is either regulated by action of the *FCGR2C* promoter or by deletion of a negative regulatory elements in the *FCGR2B* promoter. Humans with CNR1 deletion show a reduced FcyRIIIA-mediated NK cell ADCC, and FcyRIIB expression on NK cells was able to fully inhibit redirected FcyRIIC-mediated ADCC (9).

In case of FcγRIIC, CNV can only play a role in case of classic *FCGR2C*-ORF alleles, but in these cases the amount of copies of *FCGR2C*-ORF alleles is clearly linked to expression values (19, 43).

In FcγRIIIA, surface expression of FcγRIIIA on NK cells is also linked to gene copies of *FCGR3A* (63), although the expression levels are not as clearly associated as in *FCGR3B* and no significant correlation with mRNA levels could be found (43) suggesting some form of transcriptional regulation. The level of expression on NK cells is, at least for 1 vs. 2 copies, related to the level of killing of target cells in (redirected) ADCC assays (63).

## Linkage Disequilibrium at the *FCGR2/3* Locus

With all the *FCGR2* and *FCGR3* genes so closely associated in the *FCGR2/3* locus, the different SNPs and CNRs are prone to have a high degree of linkage disequilibrium (LD), and we have recently published a large overview of LD at the locus (43), which showed LD to occur across the locus. The LD found was similar to LD previously found between some selected sets of variants (98, 124, 125). In addition, the *FCGR3A*-p.66Leu/His/Arg SNP has been shown to be in LD with *FCGR3A-*p.Val176Phe (70), and this linkage was responsible for an initially observed difference in binding affinity for IgG in *FCGR3A*-p.66Leu/His/Arg (126), which was later shown to be solely the result of the linkage with the *FCGR3A*-p.Val176Phe (70), which conferred the difference in binding affinity. However, later binding assays with stratified groups of individuals showed some direct differences in binding affinity resulting from the *FCGR3A*-66Leu/His/Arg itself (71). Thus, knowledge of LD is very important for a correct interpretation of genotyping results.

Consideration of linkage disequilibrium should have a great impact on all the association studies performed for variants at this locus, which often genotype one or two variants only (mostly the *FCGR2A*-p.His166Arg and *FCGR3A*-p.Val176Phe SNPs). Associated variants found in these studies may be the mere reflection of an even stronger association for a variant that is in LD with the identified variant, but was not genotyped in the study (i.e., an increased prevalence of *FCGR3A*-p.176Val in a certain patient group may simply be the result of the classic *FCGR2C*-ORF haplotype being even more increased in the same patient group, because these two variants are in LD with each other). If only the *FCGR3A*-p.Val176Phe SNP is genotyped, this could lead to the wrong conclusion that *FCGR3A*-p.176Val causes an increased susceptibility to the disease. To be able to most accurately identify a potentially causative variant for an increased disease susceptibility, all the known functional genetic variants at the *FCGR2/3* locus have to be genotyped, as we have done with the MLPA technique in our studies. A multiple logistic regression analysis can then identify independent risk markers. Even then, for variants that are in strong LD with other variants, it may be hard to identify independently associated variants, and large groups are needed. For instance, in single logistic regression analyses we observed four variants that are in strong LD with each other, to be all associated with an increased susceptibility to Kawasaki Disease (KD) (43) as well as Immune thrombocytopenic purpura (ITP) (65). It seems likely that only one of these variants (the classic *FCGR2C*-ORF haplotype) actually causes the increased risk, but to prove this as an independent marker in a multiple logistic regression analysis, many more patients would need to be included in the association study, which is not easily done for rare diseases. On the other hand, for SNPs that are in less strong LD, multiple logistic regression analysis can still identify independent risk markers also in smaller patient groups.

Considering the strong LD and well-defined structure of CNRs at the locus, it may be better to analyze -and report on- some of the genetic variations at the locus as haplotypes, instead of analyzing single variations. This has already been the standard for the SNPs in *FCGR3B*, which are usually reported as the haplotypes NA1, NA2 and SH. Now, since we know that CNV at the *FCGR2/3* locus always occurs in CNRs, we suggest that CNV should be analyzed in the form of these CNRs, which basically form haplotypes. For instance, CNV in *FCGR3B* never occurs alone, but is always accompanied by CNV of *FCGR2C* and *HSPA7* (in essence, CNV in *FCGR3B* is in perfect LD with CNV in *FCGR2C* and *HSPA7*). Thus, an independent association of *FCGR3B* CNV with disease is impossible to prove, although in this case seems very likely because both the *FCGR2C* and *HSPA7* genes are pseudogenes in the majority of individuals. However, in case of the most frequent deletion allele of CNR1, the ectopic expression of FcyRIIB on NK cells (9, 99) may also play a role in disease susceptibility, and these effects are impossible to distinguish with genetic association studies.

Similarly, a decreased copy number of *FCGR3A*, which most often occurs as part of the copy number region CNR2, is in these cases always accompanied by the newly described *FCGR2A/2C* chimeric gene (56) (in essence, decreased copy number of *FCGR3A* is in very strong LD with the presence of an *FCGR2A/2C* chimeric gene). Concluding, the presence of CNV at the *FCGR2/3* locus cannot be analyzed separately for single genes, and therefore we think it is better to report the CNV as haplotypes in the form of the different CNRs at the locus.

From a functional point of view, *FCGR2C* variations should also be reported as haplotypes (classic *FCGR2C*-ORF, non-classic *FCGR2C*-ORF and *FCGR2C*-Stop). These consist of two (possibly three) SNPs that together determine expression of FcγRIIC (43). Analyzing the *FCGR2C*-p.Gln57Ter alone would include individuals with the non-expressed non-classic *FCGR2C*-ORF haplotype within the group of the classic *FCGR2C*-ORF haplotype, whereas we show that on a phenotypic and functional level, that the non-classic *FCGR2C*-ORF haplotype is similar to *FCGR2C*-Stop. Therefore, these *FCGR2C* variations should always be genotyped together and reported as haplotypes. *FCGR2C*-p.Gln57Ter should not be genotyped alone.

In view of the LD at the locus, and because the FcγR proteins encoded by the genes are functionally related and could thus act together in pathophysiologic mechanisms, it could even be attempted to report extended haplotypes, including all the different SNPs across the whole locus. However, the LD is not absolute for any of the SNPs, and thus the list of possible haplotypes is very long. Therefore, such an approach seems unpractical and confusing. When analyzing the locus as extended haplotypes, disease associations of a single gene encoding one of the FcγRs will not be obvious anymore, which will obscure valuable information on pathophysiology of the disease that is studied.

## Ethnic Variation at the *FCGR2/3* Locus

Several reports have shown that extensive ethnic variation exists at the *FCGR2/3* locus (43, 97, 98), especially for the *FCGR2C* haplotypes (43, 118), which is relevant for genetic association studies, as it emphasizes the importance of carefully selecting ethnicity-matched control groups.

## Evolution of the *FCGR2/3* Locus

The *FCGR2A* gene, defined as a gene that contains an ITAM within its sequence, appears to be specific to primates (127), and has evolved from its ortholog *fcgr3* in non-primate animals by NAHR and integration of a retroviral element that included the ITAM (127). The *FCGR3(A)* gene in primates is an ortholog of non-primate *fcgr4* (127), in fact, in some primate species this gene is also indicated as *FCGR4*. Finally, *FCGR2B* in primates is an ortholog of *fcgr2b* (127). Taken together, the basic structure of the *FCGR2/3* locus in primates is *FCGR2A-HSPA6-FCGR3A*(in some cases known as *FCGR4*)*-FCGR2B*, with *FCGR2A* being by far the most distinct from non-primate genes in this locus (127).

The *FCGR2C* and *FCGR3B* (and pseudogene *HSPA7*) genes appear to have evolved more recently. They were formed in a segmental duplication of the *FCGR2/3* locus that forms the structure *FCGR2A-HSPA6-FCGR3A-FCGR2C-HSPA7-FCGR3B-FCGR2B* (15).

Several studies have tried to determine the presence of this segmental duplication in other primates, with sometimes discordant results. It appears that within the great apes (Hominidae family), only the members that are closest to humans (the Homininae subfamily) have the segmental duplication, as shown in humans, chimpanzees (*Pan troglodytes*) (97, 127) and gorillas (*Gorilla gorilla*) (127), although one study suggested that even chimpanzees did not have the *FCGR2C* and *FCGR3B* genes (128). The other members of the Hominidae family, the orangutans, do not appear to have duplicated the locus (97, 127, 128). A little more distant, the gibbons may however have separate *FCGR3A* and *FCGR3B* genes (97) although this could not be replicated in the lar gibbon (*Hylobates lar*) with gene-specific primers for *FCGR2C* in a recent study (127). Regarding non-hominoid primates, evidence from all studies suggests that the *FCGR3B* gene is not present in macaque species (97, 127–130), nor in baboons (97, 130), with the rhesus macaque (*Macaca mulatta*) (97, 128–130), crab-eating macaque (*Macaca fascicularis*) (97, 127, 129) and hamadryas baboon (*Papio hamadryas*) (130) studied in detail. Confirming these genetic findings, no CD16 expression could be detected on neutrophils from macaques or baboons (130). On the other hand, neutrophils of sooty mangabey (*Cercocebus atys*) did express CD16 (130), which may suggest the presence of an *FCGR3B* gene, although direct genetic evidence for divergence of the *FCGR3* gene could not be found for mangabey (unknown species) (97). With all these studies, it must be taken into account that the complexity of the *FCGR2/3* locus may have precluded authors from finding *FCGR2C* and *FCGR3B* genes in genomes that are not so well characterized.

In any case, the segmental duplication that created the *FCGR2C* and *FCGR3B* genes appears to have occurred relatively recently in evolution and the most thorough and recent study by Lejeune et al. (127) restricts the segmental duplication to Homininae and estimates the event to have occurred <9.2 million years ago. Therefore, it is not so surprising that null variants of these genes are compatible with life in humans; *FCGR2C* is a pseudogene in >80% of the population and healthy human individuals lacking the *FCGR3B* gene have been described (122). Indeed, a complete lack of the *FCGR3B* gene was found in about 0.2% of individuals in a large cohort of >4,000 individuals (56). However, *FCGR3B* clearly has an important role in the immune system, given the fact that low copy number increases the risk of developing SLE. Apparently, the emergence of *FCGR3B* was beneficial to the species, and this resulted in the fact that the segmental duplication was maintained, and the *FCGR3B* gene has evolved to be very different from the *FCGR3A* gene in expression pattern and function. Evolutionary pressure from helminth infection may have driven this evolution, as an association between large helminth burden and variant frequency in the *FCGR3B* gene was found in human populations (97).

On the other hand, *FCGR2C* seems to be not so beneficial, and this result of the segmental duplication has since been modified by evolution to reduce its function. We assume that with the formation of *FCGR2C* by the segmental duplication of the locus (15), this gene must have been created as a bona fide receptor with an open reading frame. Subsequently, evolution seems to have selected variants that cause reduced function in this receptor through multiple ways: i.e., as a stop codon in exon3, a splice variant in intron7 that abrogates expression (9), and a 3′UTR that does not favor expression when compared with the similar 3′UTR of *FCGR2A* (56). All these changes indicate that having an active FcγRIIC has been selected against, and the classic *FCGR2C*-ORF haplotype may be the last functional remnant of the original *FCGR2C* formed in the segmental duplication. No clear benefits for having an active FcγRIIC are known except for a possible protective effect in helminth infections (97), but it does certainly predispose for certain autoimmune diseases (19, 43). The fact that it does occur at higher frequencies in the European population is intriguing, since it is extremely rare to absent in African populations (43, 118), which constitute the ancestors of the human race. One possibility is that the few classic *FCGR2C*-ORF alleles that may have been present in the European population after the migration out of Africa, were positively selected (or at least selection *against* this variant was less strong) and now represent the increased prevalence when compared to African populations. Other possibilities could be a Neanderthal origin of the classic *FCGR2C*-ORF allele or that it has been newly created in the European population by subsequent recombinations of CNR1 and CNR4, a theory which is supported by the fact that the classic *FCGR2C*-ORF alleles are actually a haplotype of multiple SNPs in intron2 and exon3 that completely resemble *FCGR2B* (40). Considering this possibility, one could predict that *FCGR2B*-Stop alleles (56) were also formed in this way, but the rarity of *FCGR2B*-Stop alleles suggests great evolutionary pressure on this variant, which may be associated with severe autoimmunity.

## Genetic Variation in *FCGR2/3* Genes: Associations with Disease

Both SNPs and CNV in *FCGR* genes have been associated with susceptibility to several auto-immune and infectious diseases. Importantly, despite the extensive linkage disequilibrium at the *FCGR2/3* locus, most studies have assessed polymorphisms in relative isolation without a broader consideration of connected variants at the locus. Table 1 provides an overview for a selection of these associations, concentrating on meta-analyses and novel associations that have not been reviewed before by Gillis et al. (3) and Bournazos et al. (131). In general, most of the studies focus on only one or two SNPs; the *FCGR2A*-p.His166Arg and *FCGR3A*-p.Val176Phe are the most studied.

### *FCGR2A*-p.His166Arg

Individuals with the variant *FCGR2A*-p.166Arg have increased susceptibility to SLE, compared to p.166His (50, 132–134), an association which remained also in a GWAS study (135). Although several studies suggest an increased risk of p.166Arg with the development of lupus nephritis (134, 136–138), conflicting evidence exists and a previous meta-analysis did not confirm an association (132).

In contrast to the association of p.166Arg variant with SLE, *FCGR2A*-p.166His is associated with development of ulcerative colitis (77, 139). Furthermore, a genome-wide association study (GWAS) has revealed *FCGR2A*-p.166His to be strongly associated with susceptibility to Kawasaki disease, a pediatric vasculitis affecting the coronary vasculature in particular (140). This association was confirmed in a GWAS (45). Finally, concerning immune thrombocytopenia, a meta-analysis ascertained an association between *FCGR2A*-p.166His and susceptibility to childhood ITP, but not adult ITP (46, 75, 141, 142). Taking the genetic associations into account, it may be speculated that a reduced function of FcγRIIA with the p.166Arg variant is associated with a failure to clear circulating immune complexes, which is a hallmark of SLE. On the other end, a relatively more active immune response, conferred by *FCGR2A*-p.166His, is associated with the development of ITP, Kawasaki disease and inflammatory bowel disease, emphasizing the intricate role of *FCGR2A* at the interface of multiple pathways leading to autoimmunity.

### *FCGR3A*-p.Val176Phe

A diallelic SNP is responsible for p.Val176Phe polymorphism in *FCGR3A*. For SLE, a recent meta-analysis established a link with disease susceptibility and p.176Phe (50), and the p.176Phe/Phe genotype is associated with development of lupus nephritis (143). In rheumatoid arthritis, *FCGR3A*-p.176Val/Val is associated with susceptibility to the disease amongst Europeans, but not Asians (76). Considering ulcerative colitis, there is a small increased susceptibility to develop the disease with the p.176Val allele (77). Finally, several studies have associated the *FCGR3A*-p.176Val/Val genotype with increased susceptibility for ITP (19, 75, 141, 142).

### *FCGR2B*-p.Ile232Thr

FcγRIIB holds a diallelic SNP that determines the p.Ile232Thr amino acid. The p.232Thr/Thr genotype has been strongly associated with SLE in numerous studies (50, 60, 62, 98, 144–146). Interestingly, the p.232Thr allele is in linkage disequilibrium with deletion of CNR1, but when assessed together both variations are independently associated with susceptibility to SLE (98).

In RA, *FCGR2B*-p.232Thr is not associated with disease susceptibility, but strongly associated with joint damage (147).

Although studies indicated that the p.232Thr allele is increased in adult ITP (148) and in chronic childhood ITP (149), a recent meta-analysis questioned this association (6 studies) (142). However, in a recent study three childhood ITP patients that were treated with IVIg and had the p.232Thr/Thr genotype failed to respond to IVIg, whereas 17 patients who carried the p.232Ile/Ile genotype and were only observed, all had a remarkable complete recovery from ITP during follow-up without any treatment (150). Such spontaneous recovery during observation at that timepoint is observed in ~20% of individuals overall. These data collectively indicate that the *FCGR2B*-p.Ile232Thr genotype may be associated with prognosis in childhood ITP.

### Classic *FCGR2C-*ORF and *FCGR2B* Promoter Haplotype 2B.4

The classic *FCGR2C*-ORF is in strong linkage disequilibrium with the *FCGR2B* promoter polymorphism 2B.4 and a third allele, *FCGR2A*-p.62Trp (43). Only few studies investigated all these variants in the included individuals, and identified relationships between variants can therefore not be distinguished between them.

In adult autoimmune diseases, there is an association of classic *FCGR2C*-ORF with ITP (19). In rheumatoid arthritis, CD32 expression on NK cells correlated with mild disease, as compared to aggressive disease (151). However, besides classic *FCGR2C*-ORF that induces FcyRIIC expression, FcyRIIB expression on NK cells from CNR1 deletions must have contributed to this picture, as not all patients with CD32 expression had an ORF allele. Unfortunately, this was not assessed. Presence of classic *FCGR2C*-ORF was also associated with susceptibility to SLE in one study (18) although this was not found in another study (11). Similarly to RA, the *FCGR2B* promoter polymorphism 2B.4 also correlated with susceptibility to SLE, and patients with this variant showed reduced autoantibody development and development of lupus nephritis (11, 57, 58). Finally, *FCGR2C* has been identified as a candidate susceptibility gene for systemic sclerosis (152).

Regarding childhood autoimmune diseases, classic *FCGR2C*-ORF confers susceptibility to childhood ITP (19, 65) as well as Kawasaki disease (43). Classic *FCGR2C*-ORF and 2B.4 correlate positively to immunomodulatory treatment with response to intravenous immunoglobulins (IVIg) in childhood ITP (65) and, for 2B.4, in Kawasaki disease (153). Moreover, in ITP, the variants are associated with a transient disease course, and negatively associate with chronic thrombocytopenia (65).

When these observations are combined, the linked variants classic *FCGR2C*-ORF and 2B.4 are associated with susceptibility to multiple autoimmune diseases. However, where assessed, they conferred a relatively mild disease phenotype and beneficial association with treatment response to IVIg. This suggests that patients without these variants have other contributing determinants to autoimmunity that confer a relatively negative impact on disease severity.

### Deletions in Copy Number Region 1 (CNR1)

Copy number variation in *FCGR3B* can arise from insertions or deletions of CNRs in the *FCGR2/3* locus, namely CNR1 and CNR4. Deletions in CNR4 are extremely rare at ~ 0.1% of the population, whereas a deletion in CNR1 is present in 8.6% of the population (56). Almost all studies investigating CNR1 have only determined *FCGR3B* CNV, but since virtually all deletions or duplications of *FCGR3B* found by these studies will result from CNV of CNR1 we have used the terms interchangeably in the next paragraphs. As said above, whether the effect driving the association reflects expression levels of FcγRIIIB or the ectopic expression of FcγRIIB on NK cells cannot be determined.

Associations between deletions of *FCGR3B* and susceptibility to adult autoimmune diseases have been found for SLE (78, 85, 98, 105, 154), ulcerative colitis (83), rheumatoid arthritis (RA) (81, 82, 155–157), ankylosing spondylitis (84), systemic sclerosis (80), primary Sjögren syndrome (SS) (79, 155), microscopic polyangiitis and Wegener's granulomatosis (85). In SLE, deletion of CNR1 is associated with a higher frequency of lupus nephritis (158). We also recently established that CNR1 deletion is associated with chronic and IVIg-resistant immune thrombocytopenia, but not with the transient form of the disease (65). Interestingly, also duplications of the *FCGR3B* gene were found to be associated with SLE and SS (155) as well as antineutrophil cytoplasmic antibody-associated systemic vasculitis (105), although this was not evident—or conflicted—by other studies (85, 154). In RA, there seemed to be an association with more rheumatoid factor (RF)-positive disease (157), which was not picked up in a smaller study (82). These effects may be modified by other susceptibility genes such as *CCL3L1* (155) or deletions, such as *ADAM3A* (154). In contrast to these systemic autoimmune disease, no association with a deletion of *FCGR3B* has been found for Graves' disease or Addison's disease (85), which suggests that these organ-specific autoimmune diseases are not influenced by *FCGR3B* copy numbers, although one study found an association of increased copies of *FCGR3B* with the a protection against the skin blistering disease Bullous Pemphigoid (86).

Recent meta-analyses confirmed the association between *FCGR3B* copy numbers and susceptibility with autoimmune diseases for low *FCGR3B* copies for SLE, Sjogren's syndrome and Wegener's granulomatosis (78, 159). These association were similar amongst Caucasians and Asians (159). For RA, evidence may be less clear (160), as previous meta-analyses had disparate results (82, 156), although the most recent meta-analysis from 2012 (82) did find an association and furthermore a recent study describing a large cohort confirmed the association (81).

Overall, deletions of *FCGR3B* as part of CNR1 are strongly associated with development of autoimmune diseases. The notion that this association is particularly pronounced for systemic, but not organ-specific autoimmune reactions, are suggestive of divergent pathomechanisms that may be triggered by CNR1 deletions and subsequently predispose to autoimmunity.

## The use of Genetic Association Studies at the *FCGR2/3* Locus

*FCGR2/3* polymorphisms are useful when investigating the role of FcγRs in human disease by means of genetic association studies. In general, such studies can suggest that FcγRs are involved in the pathophysiology of a certain disease. More specifically, association with specific FcγR genetic variations can give a more precise clue, as they may incriminate a certain cell type in the pathophysiology. The best example is the association of FcγRIIIB CNV with SLE (11, 34, 105, 161). Since FcγRIIIB is expressed almost exclusively in neutrophils, this is a strong suggestion that neutrophils are involved in the pathophysiology of SLE. All the other FcγRs are expressed on multiple cell types, and thus, associations of genetic variation in other genes than *FCGR3B* are less indicative of a specific cellular involvement in a disease. In some cases, it may be only possible to determine whether, in very general terms, the *more activating* or the *less activating FCGR* variants are associated with the disease studied, and thus gain insight on the general role of FcγRs in the pathophysiology of the disease. An overview of the more and less activating variants at the *FCGR2/3* locus is given in Table 2. Interestingly, several variants that are more activating are in LD with each other: *FCGR2A*-p.166His, *FCGR3A*-p.176Val, and the classic *FCGR2C*-ORF, although this may be ‘balanced' by the LD of the classic *FCGR2C*-ORF with the 2B.4 promoter haplotype in *FCGR2B* (43).

**Table 2:** Effects of genetic variants at the *FCGR2/3* locus on immune function.

	**Effect on**	**More immune activation**	**Less immune activation**
*FCGR2A*-p.His166Arg	Affinity for IgG	His	Arg
*FCGR3A CNV*	Expression of activating receptor	CNV > 2	CNV <2
*FCGR3A*-p.Val176Phe	Affinity for IgG	Val	Phe
*FCGR2C* haplotype	Expression of activating receptor	Classic ORF	Stop/ Non-classic ORF
*FCGR2B*-p.Ile232Thr	Strength of inhibitory signal	Thr	Ile
*FCGR2B* promotor haplotype	Expression of inhibitory receptor	2B.1	2B.4
*FCGR3B* CNV	Expression of receptor	?	?
*FCGR3B* haplotype	Phagocytosis (unknown mechanism)	NA1	NA2

*?, no clear net result on immune activation*.

Traditionally, it has been thought that activating and inhibitory FcγRs constitute an immunological balance that ensures adequate protection against pathogens, but on the other hand does not result in auto-immunity (7). Simply speaking, *FCGR2/3* genetic variation may tip this balance to either side, leading to auto-immunity when the balance is tipped toward the activating side, or leading to decreased immunity against pathogens or cancer cells when the balance is tipped toward the inhibitory side. However, this may be an over-simplification of the matter, and marked differences in *FCGR2/3* genetic variations occur between several autoimmune and autoinflammatory diseases. In KD and in immune thrombocytopenia (ITP), it is indeed the case that the more activating variants (*FCGR2C*-ORF, *FCGR3A*-p.176Val, *FCGR2A*-p.166His) are associated with disease susceptibility. However, in other autoinflammatory diseases, the less activating variants, which would be expected to tip the balance toward the inhibitory side, actually predispose to disease. This is for instance the case in SLE, which is associated with the less activating variants *FCGR2A*-p.166Arg, and the *FCGR2B* promoter haplotype 2B.4, which causes increased expression of the inhibitory FcγRIIB (10, 11). Another difference between SLE on the one hand and KD and ITP on the other hand is that SLE is associated with low copy number of *FCGR3B* (as is Sjögren syndrome, systemic sclerosis and possibly RA). Low copy number of *FCGR3B* is not associated with KD or ITP.

Clearly, autoimmunity is not necessarily associated with more activating *FCGR2/3* genetic variations, and *FCGR2/3* variants have different, sometimes opposite, effects on different autoimmune and inflammatory diseases, suggesting different pathophysiologic contributions of IgG and FcγRs between the diseases. Possibly, activating FcγRs actually protect against SLE by enabling “waste disposal” of pathogenic immune complexes involved in the disease. On the other hand, in the diseases in which activating variants are associated (KD and ITP), damage done by IgG may be exerted directly by cellular activation via FcγRs, which is enhanced in individuals with more activating variants. In the other diseases (SLE and RA), IgG does not seem to cause harm via cellular activation via FcγRs. Interestingly, the diseases in which the more activating variants are associated with susceptibility, are also the diseases in which IVIg is an effective therapy (KD and ITP), whereas IVIg is of no value in RA, Sjögren syndrome, systemic sclerosis and is possibly beneficial in SLE but this has not been well studied (162, 163), also suggesting a difference in the pathophysiological contribution of FcγRs in these diseases. An explanation for this finding may be that IVIg blocks activating FcγRs, which is beneficial in diseases in which these activating FcγRs are directly involved in pathophysiology, whereas in diseases in which activation of FcγRs does not play a role, blockade of FcγRs is not important.

Altogether, *FCGR2/3* association studies suggest that the pathophysiological mechanisms leading to SLE, RA, and Sjögren syndrome may be fundamentally different from the mechanisms leading to KD and ITP, a fact that is supported by the observation that SLE, RA and Sjögren syndrome occur much more frequently in women than in men, whereas in KD and ITP there is a slight predisposition in males.

Concluding, knowledge of *FCGR2/3* genetic variation in autoinflammatory and autoimmune diseases may increase our knowledge on the pathophysiology of these complicated and multifactorial diseases, and may be related to effectiveness of IVIg therapy.

## *FCGR2/3* Genetic Variation and Personalized Medicine

### Autoimmunity and Transplant

Perhaps the most clinically useful application of genotyping *FCGR2/3* genetic variation could be predicting response to therapy, and *FCGR2/3* polymorphisms could be of potential value in personalized medicine. There is clinical data for Kawasaki disease, childhood ITP and SLE that correlated disease outcomes as well as response to treatment are associated with *FCGR2/3* variants as well as copy number variation of CNR1 (Table 3). This suggests that it may be possible to use genetic variants to determine prognosis and potentially guide treatment decisions. A key step toward their use would be external validation as well as an investigation of their integration with existing clinical prognostic scores.

**Table 3:** Opportunities to use *FCGR2/3* locus genotyping in personalized medicine: polymorphisms and copy number variation.

**Domain**	**Disease setting**	**Clinical setting**	**Association with *FCGR2/3* genetic variation**	**References**
Autoimmune	ITP	Prognosis	2B.4 and *FCGR2C*-ORF correlate are associated with transient disease; CNR1 deletion is associated with chronic disease	(65)
Autoimmune	ITP	Treatment	2B.4 and *FCGR2C*-ORF correlate with favorable IVIg response	(65)
Autoimmune	ITP	Treatment	*FCGR3A*-p.176Val/Val is associated with favorable response to rituximab	(164)
Autoimmune	Kawasaki disease	Treatment	2B.4 correlates with favorable IVIg response	(153)
Autoimmune	SLE	Prognosis	2B.4 shows lower rate of lupus nephritis	(11)
Autoimmune	SLE	Prognosis	CNR1 deletion is associated with lupus nephritis	(158)
Autoimmune	Rheumatoid arthritis	Treatment	*FCGR3A*-p.176Val allele confers improved response to rituximab treatment (meta-analysis, 3 studies)	(165)
Autoimmune	Rheumatoid arthritis	Treatment	*FCGR2A*-p.166Arg allele is associated with favorable response to adalimumab	(166)
Cancer	Breast cancer	Treatment	*FCGR2A*-p.166His/His and *FCGR3A*-p.176Val/Val have higher response and progression-free survival with trastuzumab in HER2-positive metastatic disease	(167)
Cancer	Breast cancer	Treatment	*FCGR2A*-p.166His/His shows better pathological response and progression-free survival after trastuzumab in HER2-positive disease	(168)
Cancer	Breast cancer	Treatment	*FCGR3A*-p.176Val/Val shows improved patient outcomes and benefit from trastuzumab	(169)
Cancer	Breast cancer	Treatment	No difference after trastuzumab observed with *FCGR2A*-p.166 and *FCGR3A*-p.176 variants	(170)
Cancer	Lymphoma	Treatment	*FCGR3A*-p.176Val/Val showed better response rates to rituximab	(171)
Cancer	Lymphoma	Treatment	*FCGR3A*-p.176Val/Val showed better response rates and progression-free survival to rituximab	(172)
Cancer	Lymphoma	Treatment	Carriers of *FCGR3A*-p.176Val allele showed better response rates	(173)
Cancer	Lymphoma	Treatment	Carriers of *FCGR3A*- p.176Val allele showed better response rates	(174)
Cancer	Lymphoma	Treatment	No difference in response to rituximab with *FCGR2A*-p.166 and *FCGR3A*-p.176 variants	(175)
Cancer	Lymphoma	Treatment	No difference in response to rituximab with *FCGR2A*- p.166 and *FCGR3A*-p.176 variants	(176)
Cancer	Lymphoma	Treatment	No difference in response to rituximab with *FCGR2A*- p.166 and *FCGR3A*-p.176 variants	(177)
Cancer	CLL	Treatment	No difference in response to rituximab with *FCGR2A*- p.166 and *FCGR3A*-p.176 variants	(178)
Cancer	Colorectal carcinoma	Treatment	*FCGR2A*-p.166His/His and *FCGR3A*-p.176Val/Val show longer progression-free survival after cetuximab	(179)
Cancer	Colorectal carcinoma	Treatment	*FCGR2A*-p.166His and *FCGR3A*-p.176Val alleles show increased response rates and stable disease after cetuximab	(180)
Cancer	Colorectal carcinoma	Treatment	*FCGR2A*-p.166His/His and *FCGR3A*-p.176Phe/Phe^*^ showed higher progression-free surival rates after cetuximab	(181)
Cancer	Colorectal carcinoma	Treatment	*FCGR3A*-p.176Phe/Phe^*^ showed higher survival rates after cetuximab	(182)
Cancer	Colorectal carcinoma	Treatment	*FCGR3A*-p.176Val/Val and p.176Val/Phe show higher progression-free survival than p.176Phe/Phe after cetuximab	(183)
Cancer	Colorectal carcinoma	Treatment	*FCGR3A*-p.176Val/Val showed shorter^*^ progression-free survival compared to p.176Val/Phe or p.176Phe/Phe	(184)
Cancer	Head and neck carcinoma	Treatment	*FCGR2A*-p.166His/His and *FCGR3A*-p.176Val/Val have longer progression-free survival with cetuximab	(185)
Transplant	Liver transplant	Treatment	After rituximab, *FCGR2A*-p.166His/His shows stronger B cell suppression, bacterial infections, and poor prognosis	(186)

*CLL, chronic lymphocytic leukemia*.

* *Result is in contrary direction to other studies*.

Similar to autoimmunity, some data have suggested that patients with the higher-affinity *FCGR3A*-p.176Val allele show enhanced B cell deletion after rituximab treatment during liver transplant setting (Table 3). On the other hand, these patients may require additional immune protection with IVIg because of increased susceptibility to bacterial infections. These data suggest that *FCGR3A* genotyping could be used to potentially offer an alternative transplant immunosuppression regimenboth during and following transplantation to intervene early to prevent immunosuppression-related complications.

### Cancer Immunotherapy

Association of *FCGR2/3* genetic variation has also been extensively evaluated in monoclonal antibody therapy in cancer patients. Antibodies directed against specific tumor antigens may help in eradicating cancer cells, and this takes place in part by cellular effector mechanisms mediated by FcγRs, such as antibody-dependent cellular cytotoxicity (ADCC) and antibody-dependent cellular phagocytosis (ADCP). Thus, the efficacy of the antibody may be related to *FCGR2/3* genetic variation. MoAb therapy is costly, and since not all patients seem to benefit from it, predicting an individual patients' response could help to identify the patients likely to respond to this therapy. Extensive data points toward the fact that patients with higher-affinity *FCGR2A*-p.166His or *FCGR3A*-p.176Val alleles have an enhanced response to monoclonal antibody-mediated anti-cancer therapy amongst solid tumors of the breast, head and neck, colorectal carcinomas as well as lymphomas (Table 3). These data are supported by extensive molecular evidence. For example, the trastuzumab mediated ADCC of anti-HER2 breast cancer cells is enhanced with *FCGR2A*-p.166His and *FCGR3A*-p.176Val, compared to the other alleles (167), and in case of neutrophil-mediated ADCC for *FCGR2A*-p.166His (187). The same has been observed with NK cell rituximab-mediated ADCC to Daudi (lymphoma) cells in *FCGR3A*-p.176Val individuals (23). Collectively, these data show on a laboratory and clinical level that individuals with *FCGR2A*-p.166His and *FCGR3A*-p.176Val have enhanced responses to antibody-mediated cancer immunotherapy and genotyping may be useful to stratify treatment regimens and potentially adjust dosing to prevent side effects. Notably, some studies have shown discordant results that are in contrast with these findings (Table 3).

Currently, the variation in results do not yet allow a strategy to justify individualized treatments to be on the basis of *FCGR2/3* polymorphisms (188). When clinical use of afucosylated IgG antibodies with increased affinity for FcγRIIIA could be combined with *FCGR2/3* genotyping, the correlation with efficacy of cancer therapy may be enhanced. A major drawback of most current genotyping studies is the fact that only two SNPs are analyzed, whereas other SNPs at the locus also potentially influence treatment response rates, and the SNPs are in LD with each other. Analysis of all the SNPs and CNV, and analysis as extended haplotypes across the locus may be more useful; the complexity of the locus requires a more comprehensive assessment that includes determination of gene copy numbers, as well as classic *FCGR2C*-ORF haplotype.

## Conclusion

The *FCGR2/3* locus is a complex genetic locus with many functional genetic variants in intricate linkage. It holds many disease associations which are different, sometimes with opposite effects, between various autoimmune and autoinflammatory diseases, which may inform us on fundamental differences in pathophysiologic mechanisms. Furthermore, the locus is promising in view of genetic prediction of efficacy of therapy, especially immunotherapy in cancer, although this is currently not yet feasible. Given the complexity of the locus and inaccuracies in the current databases holding reference sequences, research on the locus could benefit from a thorough genetic analysis that sequences through the entire region and can help to establish a correct and proper reference. Such an approach has recently been explored for *FCGR3A* using long-range sequencing with Nanopore MinION technology, and allowed a complete investigation of polymorphic sites within the gene (189). In any case, to use the full potential of genetic variation at the *FCGR2/3* locus, a comprehensive analysis of all SNPs and CNVs together is warranted.

## Author Contributions

SN and DS wrote the manuscript. TK and MH edited and critically reviewed the manuscript.

### Conflict of Interest

The authors declare that the research was conducted in the absence of any commercial or financial relationships that could be construed as a potential conflict of interest.
